# Roles of glycoprotein glycosylation in the pathogenesis of an endemic osteoarthritis, Kashin–Beck disease, and effectiveness evaluation of sodium hyaluronate treatment

**DOI:** 10.3906/sag-1903-137

**Published:** 2020-06-23

**Authors:** Sen WANG, Zongqiang GAO, Huan LIU, Peilin MENG, Cuiyan WU, Mikko J. LAMMI, Xiong GUO

**Affiliations:** 1 School of Public Health, Health Science Center, Xi’an Jiaotong University; Key Laboratory of Trace Elements and Endemic Diseases, National Health Commission, Xi’an, Shaanxi P.R. China; 2 Orthopedic Department, the Second Affiliated Hospital of Xi’an Jiaotong University, Xi’an, Shaanxi P.R. China; 3 Department of Integrative Medical Biology, University of Umeå, Umeå Sweden

**Keywords:** Kashin–Beck disease, carbohydrate chain, lectin microarray, serum, saliva

## Abstract

**Background/aim:**

We aimed to explore the roles of glycoprotein glycosylation in the pathogenesis of Kashin–Beck disease (KBD), and evaluated the effectiveness of sodium hyaluronate treatment.

**Materials and methods:**

Blood and saliva were collected from KBD patients before and after the injection of sodium hyaluronate. Normal healthy subjects were included as controls. Saliva and serum lectin microarrays and saliva and serum microarray verifications were used to screen and confirm the differences in lectin levels among the three groups.

**Results:**

In saliva lectin microarray, bindings to *Sophora japonica* agglutinin (SJA), *Griffonia (Bandeiraea) simplicifolia* lectin I (GSL-I), *Euonymus europaeus* lectin (EEL), *Maackia amurensis* lectin II (MAL-II), *Sambucus nigra* lectin (SNA), *Hippeastrum* hybrid lectin (HHL), and *Aleuria aurantia* lectin (AAL) were higher in the untreated KBD patients than in the control group. Increased levels of HHL, MAL-II, and GSL-I in the untreated KBD patients discriminated them in particular from the treated ones. Jacalin was lower in the untreated KBD patients compared to the treated KBD and control groups. In serum lectin microarray, HHL and peanut agglutinin (PNA) were increased in the untreated KBD group in comparison to the control one. AAL, *Phaseolus vulgaris* agglutinin (E+L) (PHA-E+L), and *Psophocarpus tetragonolobus* lectin I (PTL-I) were lower in the untreated KBD patients compared to the treated KBD and control groups. Hyaluronate treatment appeared to normalize SNA, AAL, and MAL-II levels in saliva, and HHL, PNA, AAL, PTL-I, and PHA-E+L levels in serum. Saliva reversed microarray verification confirmed significant differences between the groups in SNA and Jacalin, in particular for GSL-I levels, while serum reversed microarray verification indicated that HHL, PNA, and AAL levels returned to normal levels after the hyaluronate treatment. Lectin blot confirmed significant differences in HHL, AAL, and Jacalin in saliva, and HHL, PNA, PHA-E+L, and AAL in serum.

**Conclusion:**

HHL in saliva and serum may be a valuable diagnostic biomarker of KBD, and it may be used as follow-up for the hyaluronate treatment.

## 1. Introduction

Kashin–Beck disease (KBD) is an endemic osteoarthritis, which is characterized by chondrocyte necrosis and apoptosis, cartilage degeneration, and extracellular matrix degradation. It is endemic in a crescent-shaped area extending from southeastern Siberia to northeastern and southwestern China. It affects approximately 690,000 people and poses a high risk to 10.584 million others residing in 366 counties within 14 provinces or autonomous regions of China, according to the 2010 Health Statistical Yearbook of China [1].

The capacity for hyaluronate synthesis is decreased in chondrocytes from patients with KBD. Exogenous hyaluronate increases its synthesis in chondrocytes, which provides the foundation for the theory that the KBD patient should be treated with an intraarticular injection of hyaluronate. Compared with the normal control group, IL-1β and TNF-α levels were higher in the KBD group, while their expression levels decreased after administration of hyaluronate [2]. The levels of type II collagen and aggrecan mRNAs were also lower in the KBD group. After the administration of different doses of hyaluronate (100 and 500 mg/mL), their levels were significantly increased; 500 mg/mL treatment led to a more significant effect [3]. Intraarticular injections of sodium hyaluronate effectively reduced knee pain caused by KBD to a baseline value measured by using the Western Ontario and McMaster Universities Osteoarthritis (WOMAC) index [4].

Protein glycosylation plays a key role in a number of biological processes, such as development, maintenance of normal cellular functions, intercellular signaling, protein folding, protein–protein interactions, cellular differentiation, metabolism, and bacterial infections. About half of human proteins are considered to be modified with different glycosylation patterns [5]. Lectins are carbohydrate-binding proteins which have been used to discriminate carbohydrate structures, including glycosaminoglycans, glycolipids, and glycoproteins in a high-throughput manner based on slight structural differences [6]. Oligosaccharides, which exist in human saliva, include amino sugars, galactose, sialic acids (Sia), xylose, fucose, and mannose. Proteins, which perform many diverse functions, are modified by the glycan parts of glycoproteins [7]. A lectin microarray is one of the main ways to investigate variable glycosylation, as it can test many different glycan bindings simultaneously [8]. 

The etiology of KBD remains unclear. Its pathogenesis has not yet been fully defined. Currently, there are no diagnostic or treatment follow-up indicators available for KBD based on glycoprotein levels. In this study, we have suggested that the glycosylation change in the salivary and serous glycoproteins may involve the pathogenesis of KBD, and may play a role in guiding treatment. Therefore, we aimed at revealing whether such glycosylation pattern differences are present in the saliva and serum of KBD patients, and to investigate the role of knee joint injection of sodium hyaluronate on the glycosylation in the KBD patients. Lectin microarray was used to detect the expression patterns of glycosylations. Reversed microarrays and lectin blot were used to validate the results of lectin microarray. Finally, this study evaluated whether the identified potential lectins could be effective as diagnostic biomarkers for KBD, and useful in the follow-up of sodium hyaluronate treatment of KBD. 

## 2. Materials and methods

### 2.1. Whole saliva and venous blood samples collection 

Patients with KBD (n = 35; 20 males, 15 females, 61 ± 5 years old) and normal control individuals (n = 38; 20 males, 18 females, 60 ± 6 years old) were recruited from Linyou and Yongshou counties in Shaanxi Province, China. All samples were excluded if the persons showed genetic indications of bone or cartilage diseases, osteoarthritis, or rheumatoid arthritis. Sodium hyaluronate (10 mg/mL, 2 mL) was injected into a knee joint of each patient with KBD. Both venous blood and saliva were collected from the KBD patients before and 2 weeks after the injection (untreated KBD group, n = 35; treated KBD group, n = 35). Samples were also collected from the matched normal group (n = 38). At least 2 h after the last intake of food, 3 mL of peripheral blood and 3 mL of unstimulated saliva were collected; 0.9% saline was used to rinse participant’s mouth before saliva was collected. Protease inhibitor cocktail (1 µL/mL of saliva; Sigma, Darmstadt, Germany) was added to the saliva immediately after collection. The saliva was then centrifuged at 12,000 rpm, 30 min, 4 °C. The supernatant was stored at –80 °C. Venous blood samples were incubated at room temperature for 30 min. The serum was collected by centrifugation at 3000 rpm for 5 min and then used immediately or stored at –80 °C. 

Informed consent was obtained from each subject involved in the investigation. All procedures performed in the study involving human participants were in accordance with the ethical standards of the Declaration of Helsinki as revised in 1964. The study has the ethical approval of the Ethics Management Committee of Xi’an Jiaotong University (No: 2019-639).

### 2.2. Saliva and serum lectin microarrays

**Figure 1:**
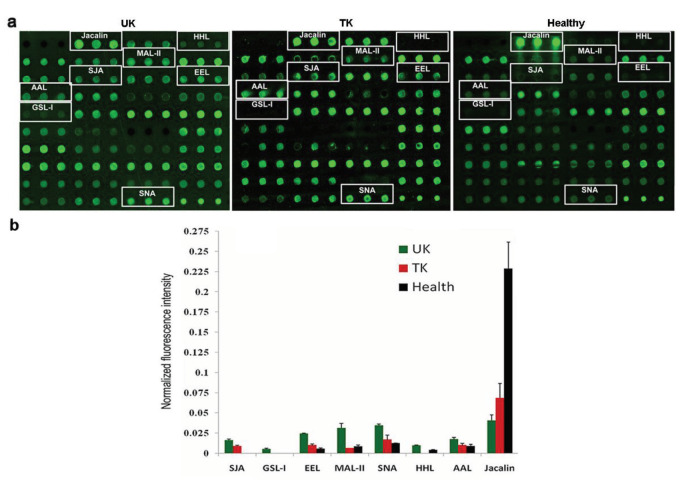
Layout of 37 lectins, the negative control (bovine serum albumin, BSA), and a positive marker contained in saliva or serum lectin microarray.

Twenty samples of saliva or serum from both the untreated and the treated KBD groups were applied to the lectin microarray. In addition, 20 samples from the control group were also applied to the lectin microarray. Samples from the untreated KBD, the treated KBD, and normal groups were divided into subgroups by age (±5 years) separately. The samples in each subgroup were pooled into one sample separately. Pooled samples were then matched according to sex. Saliva and serum lectin microarrays were produced separately and incorporated 37 lectins (Sigma, Darmstadt, Germany), which bind N- or O-linked glycans (shown in Table 1). The arrangement of the lectin microarray is shown in Figure 1. In brief, 37 lectins were dissolved in the manufacturer’s recommended buffer and then spotted on the homemade epoxysilane-coated slides with Stealth microspotting pins (SMP-10B; TeleChem, Sunnyvale, CA, USA) with a Capital smart microarrayer (CapitalBio, Beijing, China). Every lectin was spotted in triplicate on one slide. The protein labeled with Cy3 was diluted in 0.5 mL buffer containing 2% glycine, bovine serum albumin (BSA), and 0.1% Tween-20. The mixture was then used in the lectin microarrays. Finally, incubation was performed in the chamber at 37 °C for 3 h [9].

**Table:** The structures of the carbohydrate chains recognized especially by the lectins.

Lectin	Full name of the lectin	Specificity carbohydrate chain
ACA	Amaranthus caudatus	Galβ1-3GalNAcα-Ser/Thr (T antigen), sialyl-T(ST) tissue staining patterns are markedly different than those obtained with either PNA or Jacalin
AAL	Aleuria aurantia lectin	Fucα1-6 GlcNAc(core fucose), Fucα1-3(Galβ1-4)GlcNAc
BPL	Bauhinia purpurea lectin	Galβ1-3GalNAc, Terminal GalNAc
BS-I	Bandeiraea simplicifolia	α-Gal, α-GalNAc, Galα-1,3Gal, Galα-1,6Glc
ConA	Canavalia ensiformis	High Mannose, Manα1-6(Manα1-3)Man, αMannose, αGlc
DBA	Dolichos biflorus agglutinin	αGalNAc, Tn antigen, GalNAcα1-3((Fucα1-2))Gal (blood group A antigen)
DSA	Datura stramonium	(GlcNAc) 2-4, polyLacNAc and LacNAc (NA3, NA4)
ECA	Erythrina cristagalli	Galβ-1,4GlcNAc (type II), Galβ1-3GlcNAc (type I)
EEL	Euonymus europaeus lectin	Galα1-3(Fucα1-2)Gal (blood group B antigen)
GNA	Galanthus nivalis	High-Mannose, Manα1-3Man
GSL-I	Griffonia (Bandeiraea) simplicifolia lectin I	αGalNAc, αGal, anti-A and B
GSL-II	Griffonia (Bandeiraea) simplicifolia lectin II	GlcNAc and agalactosylated tri/tetra antennary glycans
HHL	Hippeastrumhybrid lectin	High-Mannose, Manα1-3Man, Manα1-6Man, Man5-GlcNAc2-Asn
Jacalin	Artocapus integrifolia	Galβ1-3GalNAcα-Ser/Thr(T), GalNAcα-Ser/Thr(Tn), GlcNAcβ1-3-GalNAcα-Ser/Thr(Core3), sialyl-T(ST). not bind to Core2, Core6, and sialyl-Tn (STn)
LCA	Lens culinaris agglutinin	α-D-Man, Fucα-1,6GlcNAc, α-D-Glc
LEL	Lycopersicon esculentum (tomato) lectin	(GlcNAc)n, high mannose-type N-glycans
LTL	Lotus tetragonolobus lectin	Fucα1-3Galβ1-4GlcNAc, Fucα1-anti-H blood group specificity
MAL-I	Maackia amurensis lectin I	Galβ-1,4GlcNAc
MAL-II	Maackia amurensis lectin II	Siaα2-3Galβ1-4Glc(NAc)/Glc, Siaα2-3Gal, Siaα2-3, Siaα2-3GalNAc
MPL	Maclura pomifera lectin	Galβ1-3GalNAc, GalNAc
NPA	Narcissus pseudonarcissus lectin	High-Mannose,Manα1-6Man
PHA-E+L	Phaseolus vulgaris agglutinin (E+L)	Bisecting GlcNAc, bi-antennary N-glycans, tri- and tetra-antennary complex-type N-glycan
PWM	Phytolacca americana	(GlcNAc)n and polyLacNAc
PSA	Pisum sativum agglutinin	Fucα-1,6GlcNAc,α-D-Man, α-D-Glc
PHA-E	Phaseolus vulgaris agglutinin (E)	Bisecting GlcNAc, biantennary complex-type N-glycan with outer Gal
PTL-I	Psophocarpus tetragonolobus lectin I	GalNAc, GalNAcα-1,3Gal, GalNAcα-1,3Galβ-1,3/4Glc
PTL-II	Psophocarpus tetragonolobus lectin II	Gal, blood group H, T-antigen
PNA	Peanut agglutinin	Galβ1-3GalNAcα-Ser/Thr(T)
RCA120	Ricinus communis agglutinin I	β-Gal, Galβ-1,4GlcNAc (type II), Galβ1-3GlcNAc (type I)
SJA	Sophora japonica agglutinin	Terminal in GalNAc and Gal, anti-A and anti-B human blood group
STL	Solanum tuberosum (potato) lectin	trimers and tetramers of GlcNAc, core (GlcNAc) of N-glycan, oligosaccharide containing GlcNAc and MurNAc
SBA	Soybean agglutinin	α- or β-linked terminal GalNAc, (GalNAc)n, GalNAcα1-3Gal, blood-group A
SNA	Sambucus nigra lectin	Sia2-6Gal/GalNAc
UEA-I	Ulex europaeus agglutinin I	Fucα1-2Galβ1-4Glc(NAc)
VVA	Vicia villosa lectin	terminal GalNAc, GalNAcα-Ser/Thr(Tn), GalNAcα1-3Gal
WFA	Wisteria floribunda lectin	terminating in GalNAcα/β1-3/6Gal
WGA	Triticum vulgaris	Multivalent Sia and (GlcNAc)n

### 2.3. Statistical analysis of saliva and serum lectin microarrays

Three replicate slides of each sample were consistently analyzed. We obtained primary fluorescence intensity value of each lectin, then each value was globally normalized by getting the ratio of each intensity value compared with the sum of 37 lectin values. The mean of 3 replicate ratios was the final fluorescence intensity value of each lectin. Subsequently, we separately compared the means of paired groups (untreated KBD/treated KBD, untreated KBD/normal, and treated KBD/normal). Fold changes ≥1.5 or ≤0.67 between the pairs indicated up- or down-regulation of each lectin, respectively.

### 2.4. Saliva and serum reversed microarrays verification

A reversed microarray analysis was used to verify the lectin microarray results. Salivary and serous protein samples from 15 untreated KBD patients, 15 KBD patients treated with sodium hyaluronate, and 18 normal individuals were spotted in triplicate on the microarray. The Cy3-labeled lectins were applied to detect the specific glycan structures in the salivary and serous protein samples, which were immobilized on the slides. Lectins GSL-I, SNA, and Jacalin were selected for saliva microarrays, and HHL, AAL, and PNA for serum microarrays. The slides were scanned using a Genepix 4000B confocal scanner, and the acquired images were analyzed at 532 nm to detect Cy3.

### 2.5. Statistical analysis of saliva and serum microarrays verification

Significant differences between the untreated KBD, the treated KBD, and the normal groups were calculated using one-way ANOVA with SPSS Statistics 19 (IBM, Armonk, NY, USA). Differences were considered statistically significant for values of P < 0.05.

### 2.6. Lectin blot analysis

In order to further confirm the different abundance of significant glycans, the SDS-PAGE and lectin blotting analysis was performed with 3 lectins (HHL, AAL, and Jacalin)in pooled saliva group samples and 4 lectins (HHL, PNA, PHA-E+L, and AAL) in both pooled saliva group samples and pooled serum group samples of the normal control, the untreated KBD, and the treated KBD groups separately. In brief, the samples were run on a 10% SDS-PAGE polyacrylamide resolving gel and a 3% stacking gel. Afterwards, the proteins in the gels were transferred to a PVDF membrane (MilliporeSigma, Burlington, MA, USA) with a wet transfer unit (Hoefer Scientific, Holliston, MA, USA) at 100 V for 1.5 h. The membrane was washed twice with TTBS (10 mmol/L Tris-HCl, 150 mmol/L NaCl, 0.05% Tween-20, pH 7.5) and blocked with Carbo-Free Blocking Solution (Vector, Burlingame, CA, USA) for 1 h at room temperature. The membrane was incubated with Cy5-labeled (GE Healthcare, Buckinghamshire, UK) lectins (2 mg/) at 4 °C overnight in the dark. Finally, the membrane was scanned using a phosphorimager (Molecular Dynamics Inc., Sunnyvale, CA, USA) [9].

## 3. Results 

### 3.1. Differential lectins in saliva and serum lectin microarrays

**Figure 2:**
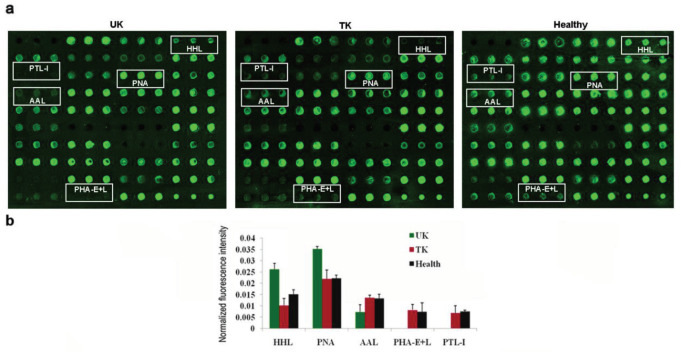
Salivary glycoprotein glycosylation patterns determined with Cy3-labeled lectins spotted onto a lectin microarray. UK: untreated KBD patients; TK: treated KBD patients; Healthy: normal controls. a. Profiles of Cy3-labeled salivary proteins from the UK, the TK, and the healthy individuals bound to the lectin microarrays. The lectin microarrays revealed significant lectins marked with white rectangles. b. Significant differences in the lectin levels between the three groups. The bars show mean ± SD of three biological replicates from each group.

Saliva lectin microarray showed significant differences in the fluorescence intensities of 8 lectins between the untreated KBD, the treated KBD, and the normal groups (Figure 2). Stronger signals for SJA, GSL-I, EEL, MAL-II, SNA, HHL, and AAL were observed in the saliva from the untreated KBD patients than from the treated patients with KBD and the control group. Meanwhile, Jacalin signal was lower in the saliva from the untreated KBD patients and the treated KBD group in comparison with the control group. After hyaluronate treatment, the levels of SNA, AAL, and MAL-II appeared to return to approximately normal levels.

**Figure 3:**
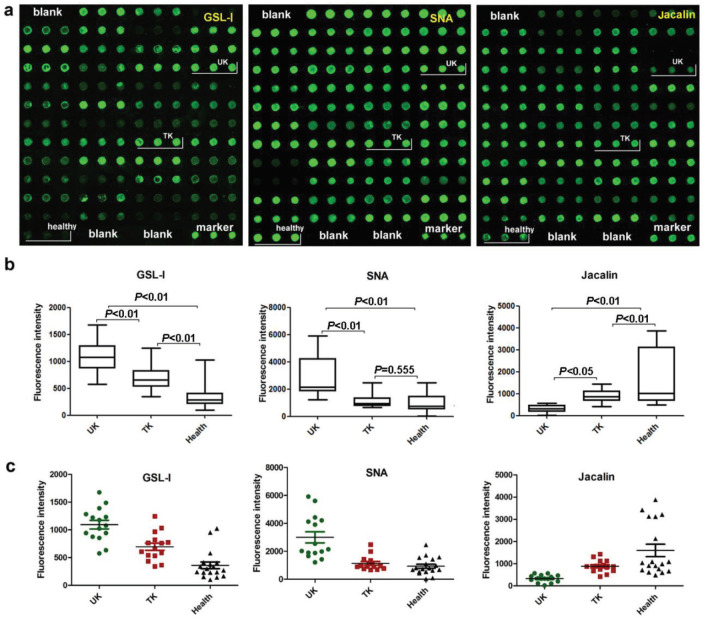
a. Serous glycoprotein expression levels were determined with Cy3-labeled lectins using a lectin microarray. UK: untreated patients with KBD; TK: treated patients with KBD; Healthy: normal controls. The lectin microarrays revealed significant lectins marked with white rectangles. b. Significant differences in the lectin levels between the three groups. The bars show mean ± SD of three biological replicates from each group.

In the serum lectin microarray (Figure 3), significant differences in the fluorescence intensities of 5 lectins were observed between the untreated KBD, the treated KBD, and the control groups. Strong signals of HHL and PNA were typical for the samples of the untreated KBD group in comparison to the normal ones. Meanwhile, decreased AAL, PHA-E+L, and PTL-I signals were observed in the serum samples of the KBD patients. Hyaluronate treatment appeared to normalize the levels of HHL, PNA, AAL, PHA-E+L, and PTL-I.

### 3.2. Differential lectins in saliva and serum microarrays verification

**Figure 4:**
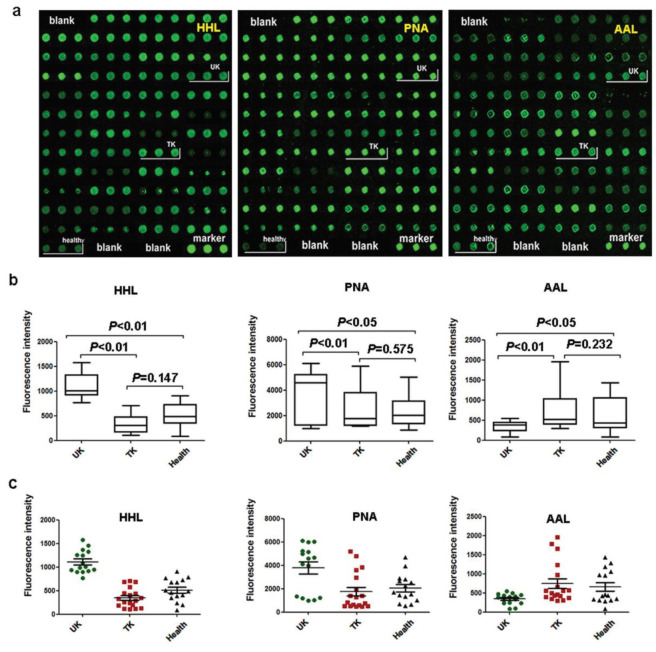
Verification of the differences in the saliva levels of the three lectins GSL-1, SNA, and Jacalin. UK: untreated patients with KBD; TK: treated patients with KBD; Healthy: normal controls. a. Scanned pictures of Cy3-labeled lectins bound to the salivary protein microarrays. b. Box plot analysis of the salivary microarray data obtained from the three groups. Error bars show 95% confidence intervals for mean values. The P-values indicate the statistical significance of differences between the groups. c. Scatter plot analysis of the salivary protein microarray data obtained from the three groups. Lines show mean ± SEM.

Salivary protein microarray showed significantly stronger GSL-I signal in the untreated KBD patients (Figure 4). Significantly elevated SNA signal levels in the untreated patients were normalized by the hyaluronate treatment, although the level still remained higher than in the control group (Figure 4). Saliva from the untreated patients had significantly lower levels of Jacalin than the healthy controls (Figure 4).

**Figure 5:**
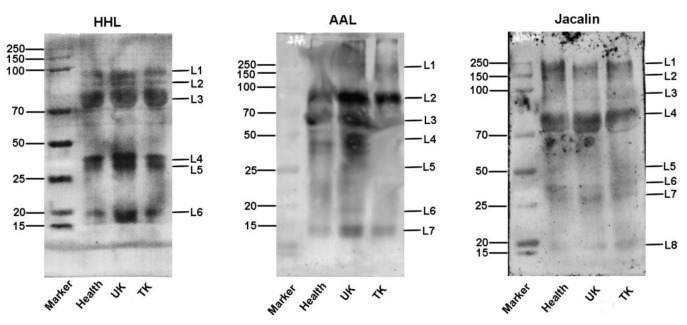
Verification of the differences in the sera levels of the three lectins HHL, PNA, and AAL. UK: untreated patients with KBD; TK: treated patients with KBD; Healthy: normal controls. a. Scanned pictures of Cy3-labeled lectins bound to the serous protein microarrays. b. Box plot analysis of the serous microarray data obtained from the three groups. Error bars show 95% confidence intervals for mean values. The P-values indicate the statistical significance of differences between groups. c. Scatter plot analysis of the serous protein microarray data obtained from the three groups. Lines show mean ± SEM.

Data from serum microarray verification showed that significantly increased levels of HHL and PNA signals in the untreated KBD group declined to the control level in the treated group (Figure 5). A significantly decreased level of AAL signal in the untreated KBD group increased to the control level in the treated group (Figure 5).

### 3.3. Lectin blot analysis

**Figure 6:**
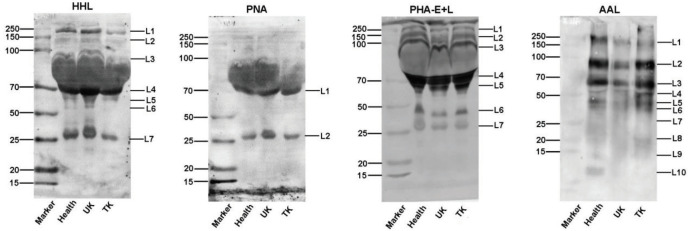
Lectin blot analysis of the differential expressions of the glycopatterns in the saliva from three groups: Healthy: normal control; UK: untreated KBD; TK: treated KBD. The three groups of saliva pooled samples using 3 lectins (HHL, AAL, and Jacalin). The apparent bands belong to different molecular weights ranging from 15 to 250 kDa, which are marked as L1–L8, respectively.

**Figure 7:**
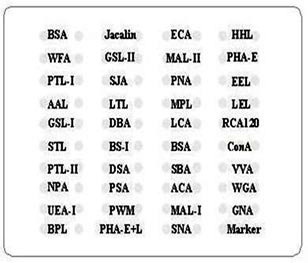
Lectin blot analysis of the differential expressions of the glycopatterns in the sera from three groups: Healthy: normal control; UK: untreated KBD; TK: treated KBD. The three groups of saliva pooled samples using 4 lectins (HHL, PNA, PHA-E+L, and AAL). The apparent bands belong to different molecular weights ranging from 15 to 250 kDa, which are marked as L1–L10, respectively.

The different abundance of glycans among normal control (healthy), the untreated KBD (UK), and the treated KBD (TK) groups were confirmed by the lectin blotting analysis with 3 lectins (HHL, AAL, and Jacalin) in saliva samples and 4 lectins in serum samples (HHL, PNA, PHA-E+L, and AAL). The results of the lectin blotting analysis showed apparent bands belonging to different molecular weights ranging from 15 to 250 kDa. The apparent bands were marked as L1–L10 (Figures 6 and 7). 

Among the selected lectins, HHL showed stronger binding to 2 bands between 70 and 100 kDa (L1, L2), 2 bands between 25 and 50 kDa (L4, L5), and 1 band between 15 and 20 kDa (L6) in the saliva of the UK group than in the TK group, and also showed stronger binding in the TK group than in the healthy group. 

AAL showed stronger binding to one band between 15 and 20 kDa (L6) in the saliva of the UK group than to the TK and the healthy groups. On the contrary, Jacalin exhibited weaker binding to 2 bands between 100 and 250 kDa (L1, L2) and 1 band between 25 and 50 kDa (L6) in the saliva of the UK group than in the TK group, and also showed weaker binding in the TK group than in the healthy group (Figure 6). 

HHL showed stronger binding to one band 70 kDa (L4) in the serum of the UK group than in the TK and healthy groups. PNA showed stronger binding to one band 70 kDa (L1) in the serum of the UK group than in the TK and healthy groups. On the contrary, PHA-E+L exhibited weaker binding to one band 50–70 kDa (L5) in the serum of the UK group than in the TK and healthy groups. AAL also exhibited weaker binding to one band 70–100 kDa (L2) and one band 50–70 kDa (L3) in the serum of the UK group than in the TK and healthy groups (Figure 7). The results were coincident with the results from the lectin microarrays.

## 4. Discussion

Glycosylation is one of the most common posttranslational modifications of secreted proteins and plays a significant role in cell–cell interactions, cell adhesion, malignant transformation, and metastasis [10]. It also has an important role in the molecular recognition events of the body. The glycosylation patterns of glycoproteins provide clues about cell metabolism and the expression and function of oligosaccharides. Glycoproteins can exist in many glycosylated variants, and the distribution of different classes of oligosaccharide structures is most often specific for each glycosylation site [11].

Although serum is frequently used as a sample to analyze various biomarkers, saliva is also a good indicator of the plasma levels of various substances. Although both saliva and serum are samples from systemic sources, the components derived from tissues such as cartilage can accumulate in them. The extracellular matrices of tissues such as the cornea, hyaline cartilage, and nucleus pulposus of the intervertebral disks are rich sources of glycoproteins and proteoglycans (PGs) [12]. More importantly, shifts in the glycosylation patterns have been identified in diseases, such as in IgG of the serum of rheumatoid arthritis patients [13], which was later found to be responsible for the activation of the complement system [14]. Lectins can be valuable tools in the diagnosis of diseases, and even in discrimination of their stages [15], although the actual protein with altered glycosylation and/or its function would not be known. 

In this study, the goal was to identify changes in glycosylation patterns in KBD by using a group of lectins. Hyaluronate treatment has been reported to alleviate the symptoms of KBD patients [4]. Therefore, we also screened whether the treatment would reveal lectin levels, which are altered in KBD but normalized by hyaluronate therapy, since they could be useful in evaluating the efficacy of the treatment. As a result, the first screening experiments identified several lectins which had differential expression levels in the KBD saliva and serum in comparison to the normal samples. Three of those lectins (GSL-I, SNA, and Jacalin) were selected for the verification microarray of saliva samples, and three were selected for serum samples (HHL, PNA, and AAL). The carbohydrate chain Galα1-3-Gal is recognized by PNA, high mannose is recognized by HHL, and GlcNAc is recognized by AAL, Jacalin, and PHA-E+L.

The glycosylation pattern changes can have marked effects in the functions of proteins. Previously, the levels of the Galα1,3-Gal antigen reduced in HT-transgenic porcine cartilage conferred resistance to delayed rejection [16]. Expression levels of high mannose-type N-glycans were significantly increased at the later stages of mouse chondroprogenitor cell differentiation. On the other hand, the levels of some high mannose-type N-glycans were recently shown to be significantly decreased in both human OA cartilage and degraded mouse cartilage [17,18].

In developing rat embryos, the presence of numerous lectin binding affinities has been associated with a general reduction of oligosaccharide structures during development [19]. Primary OA chondrocytes are characterized by significantly higher levels of high mannose-type N-glycans and sialic acid-capped N-glycans compared to chondrocyte cell lines. In immortalized chondrocyte cell lines, the patterns of O- and N-linked oligosaccharides appear to be shifted toward reduced levels of high mannose-type and sialic acid-capped N-glycans, as well as increased levels of fucosylated O-glycosylation products [20]. An alteration in high mannose-type N-glycans has been attributed to the expression of the Mgat1 gene, which regulates the expression of the key proteases matrix metalloprotease-13 and aggrecanase-2 during the cartilage degradation process [18]. Additionally, the levels of high mannose-type N-glycans in primary human chondrocytes decrease in response to inflammatory cytokines IL-1β and TNF-α [21]. The GlcNAc-accelerated production of hyaluronate is associated with the induction of hyaluronate synthase-2, a key enzyme involved in hyaluronate synthesis [22].

Intraarticular hyaluronate injection has been proposed to have many therapeutic mechanisms of action in the OA knee, including antiinflammatory effects, joint lubrication, shock absorption, chondroprotection, proteoglycan synthesis, and cartilage matrix alteration. At present, there are no good indicators for how to observe the biological effects of the treatment. Lectin analysis may be a potential assay for the follow-up of hyaluronate treatment, not only for KBD, but also for other forms of arthritis. In both the saliva and serum samples, HHL signal was stronger in the patients with KBD than in the normal controls and normalized after hyaluronate treatment. HHL binding was the best marker to differentiate the KBD patients from the controls. It also differentiated between the untreated and the hyaluronate-treated groups and, thus, might be a valid biomarker to evaluate the efficacy of the treatment. We plan to analyze the lectin levels in chondrocytes and to confirm diagnostic biomarkers of KBD in the next step. The method used to reveal the differences in the glycopatterns of human salivary and serous glycoproteins may provide pivotal information to help researchers understand other bone joint diseases. The selected lectins can only indicate changes in overall glycosylation patterns, but do not identify specific proteins involved. Therefore, the effects of these changes on protein functions cannot be defined by these analyses. However, it does not reduce the value which this type of assay can have in the diagnosis of KBD.

## Acknowledgments

This study was supported by the National Natural Science Foundation of China (grant numbers: 81472924 and 81502766); National Natural Science Foundation of China key international cooperation projects (81620108026).

## Contribution of authors

Sen WANG and Zongqiang GAO contributed equally to this work.
